# Metal-Nano-Ink Coating for Monitoring and Quantification of Cotyledon Epidermal Cell Morphogenesis

**DOI:** 10.3389/fpls.2021.745980

**Published:** 2021-09-21

**Authors:** Kotomi Kikukawa, Kazuki Yoshimura, Akira Watanabe, Takumi Higaki

**Affiliations:** ^1^Graduate School of Science and Technology, Kumamoto University, Kumamoto, Japan; ^2^Faculty of Science, Kumamoto University, Kumamoto, Japan; ^3^Institute of Multidisciplinary Research for Advanced Materials, Tohoku University, Sendai, Japan; ^4^International Research Organization for Advanced Science and Technology, Kumamoto University, Kumamoto, Japan

**Keywords:** *Arabidopsis thaliana*, machine learning-based cell segmentation, metallographic microscopy, pavement cell morphogenesis, quantitative evaluation of cell shapes

## Abstract

During cotyledon growth, the pavement cells, which make up most of the epidermal layer, undergo dynamic morphological changes from simple to jigsaw puzzle-like shapes in most dicotyledonous plants. Morphological analysis of cell shapes generally involves the segmentation of cells from input images followed by the extraction of shape descriptors that can be used to assess cell shape. Traditionally, replica and fluorescent labeling methods have been used for time-lapse observation of cotyledon epidermal cell morphogenesis, but these methods require expensive microscopes and can be technically demanding. Here, we propose a silver-nano-ink coating method for time-lapse imaging and quantification of morphological changes in the epidermal cells of growing *Arabidopsis thaliana* cotyledons. To obtain high-resolution and wide-area cotyledon surface images, we placed the seedlings on a biaxial goniometer and adjusted the cotyledons, which were coated by dropping silver ink onto them, to be as horizontal to the focal plane as possible. The omnifocal images that had the most epidermal cell shapes in the observation area were taken at multiple points to cover the whole surface area of the cotyledon. The multi-point omnifocal images were automatically stitched, and the epidermal cells were automatically and accurately segmented by machine learning. Quantification of cell morphological features based on the segmented images demonstrated that the proposed method could quantitatively evaluate jigsaw puzzle-shaped cell growth and morphogenesis. The method was successfully applied to phenotyping of the *bpp125* triple mutant, which has defective pavement cell morphogenesis. The proposed method will be useful for time-lapse non-destructive phenotyping of plant surface structures and is easier to use than the conversional methods that require fluorescent dye labeling or transformation with marker gene constructs and expensive microscopes such as the confocal laser microscope.

## Introduction

The cotyledon pavement cells of most dicotyledonous plants have a simple rectangular shape just after germination, but drastically change into a jigsaw puzzle-like shape with waving lateral cell walls as the leaf expands (Higaki et al., 2016, 2017). The physiological significance of the jigsaw puzzle-like morphology has not been fully elucidated, although it has been suggested that it may have a role in strengthening the leaf surface (Glover, 2000), stomatal spacing (Glover, 2000; Higaki et al., 2020), organ-level morphogenesis (Kierzkowski et al., 2019; Eng et al., 2021), and reducing mechanical pressure on the cell wall from inside the cell (Sapala et al., 2018). Time-lapse observation is a fundamental and important technique for studying temporal changes of the epidermal cell morphology. The conventional replica and fluorescent labeling methods have been commonly used for time-lapse observation of cotyledon epidermal cells. In the replica method, a silicon polymer is pressed onto the leaf surface to copy the cell shapes (Elsner et al., 2012; Borowska-Wykręt et al., 2013). It is relatively easy to observe cell shapes over time with this method, but it possibly causes contact damage to the cotyledon with each observation. The most common technique is fluorescent labeling, in which fluorescent proteins or dyes are used to label cell walls or plasma membranes (Zhang et al., 2011; Armour et al., 2015; Kierzkowski et al., 2019; Higaki and Mizuno, 2020; Seerangan et al., 2020; Eng et al., 2021). However, transformation is required when using fluorescent proteins, and therefore the plant species that can be studied with this method are limited. In the case of vital staining with fluorescent dyes, it is difficult to maintain the localization of fluorescent dyes during long-term observation over several days, and the cytotoxicity of the dyes may become a serious problem. In addition, phototoxicity due to the laser light irradiation required for fluorescence excitation should be considered even though the development of fluorescence probes with low toxicity has progressed (Uno et al., 2021; Yagi et al., 2021). Furthermore, the acquisition of sufficiently high-quality images to quantitatively evaluate the cell shapes requires an expensive confocal laser microscope and skill in its use. Considering these technical limitations, we tried to develop an easy and versatile method to monitor and quantitatively evaluate the changes in cotyledon epidermal cell shapes.

In this study, we propose an observation system for quantitative analysis of epidermal cell morphogenesis in growing *Arabidopsis thaliana* cotyledons using a metal nanoparticle ink (metal nanoparticle dispersion solution; hereafter referred to as metal ink) coating (Zhuo et al., 2020) and a metallographic microscope. Metallographic microscopy uses reflected light to observe the surface of metal materials. Although it is mainly used in engineering and not commonly used in biology, it requires less skill than confocal laser microscopy. In addition, metallographic microscopes are inexpensive and have low installation costs compared with high-spec microscopes for biological research, such as confocal laser and light sheet microscopes. Unlike fluorescently stained images of cell walls or plasma membranes, our metallographic images contain a lot of information other than the epidermal cell contours, although these could be easily and accurately extracted by automatic cell segmentation based on deep learning. This machine learning-based cell segmentation technique allowed us to easily perform quantitative evaluation of epidermal cell morphology from our metallographic images. Here, we provide details of our proposed method and demonstrate its usefulness using examples of cotyledon epidermal cell morphometry with the triple knockout mutant of the basic Pro-rich protein (BPP) microtubule-associated protein family members *bpp125*, which shows severely abnormal jigsaw puzzle-type cell morphogenesis (Wong et al., 2019). Using our proposed system, we successfully analyzed the cotyledon epidermal cell shapes and morphogenesis without the need for fluorescent labeling and a high-spec microscope.

## Materials and Methods

### Plant Materials

Seeds of the *A. thaliana* wild type (Col-0), *bpp125* triple mutant (Wong et al., 2019), and the transgenic line expressing green fluorescent protein-tagged plasma membrane intrinsic protein 2a (GFP-PIP2a), which is a plasma membrane marker (Cutler et al., 2000), were sown in culture soil (Jiffy-7; Sakata Seed Corp., Kanagawa, Japan) in plastic pots and grown in a growth chamber (LH-241PFP-S; NK system, Osaka, Japan) at 23.5°C under a 16-h light/8-h dark cycle. The light intensity during the light period was 86.2 μmol^–1^ m^–2^. Seeds of carrot (*Daucus carota*) (Nichinou, Nagano, Japan), petunia (*Petunia hybrida*) (Nichinou), Japanese white radish (*Raphanus sativus*) (Nichinou), and snapdragon (*Antirrhinum majus*) (Nichinou) were grown under the same conditions as the *A. thaliana* seeds.

### Preparation of Silver Ink Solution

Silver nanoparticle cyclohexanone dispersion ink (INAg 30–50 CHX; IOX, Osaka, Japan; average particle size 30 nm; hereafter referred to as silver ink) was used. First, 10 μl of silver ink stock solution (silver concentration 50 wt%) was diluted two times by adding an equal volume of distilled water in a microtube and pipetted. Then, the solution was sonicated for 10 min using an ultrasonic cleaner (SWT710; Citizen, Tokyo, Japan). The twofold dilution with distilled water and 10-min sonication procedure was repeated to prepare eightfold diluted silver ink solution. Next, 2 μl of the eightfold diluted silver ink was diluted to 160-fold (0.31 wt%) by adding 38 μl of distilled water, followed by pipetting and sonication for 10 min. This final silver ink solution was not stored, but prepared each time it was applied to the plant samples.

### Metallographic Microscopy

A metallographic microscope (BX53M; Olympus, Tokyo, Japan) equipped with a CMOS camera (DP74; Olympus) was used for the observations. The cotyledons of the seedlings were placed on a biaxial goniometer (GN2; Thorlabs, Newton, NJ, United States) and observed with a 20 × objective lens [MPlan FL N × 20; Olympus; numerical aperture (NA) = 0.45]. The microscope imaging software cellSens (Olympus) was used for image acquisition. The imaging conditions were 50% LED power, 610 μs exposure time, and “Low” contrast. The instant EFI (Extended Focus Image) function of the cellSens software was used to acquire omnifocal images.

### Examination of Illumination Conditions Using Coins

We used the Japanese five-yen coin coated with the silver ink (50 wt%) as a standard for metallographic microscopy. The surfaces were observed under bright-field and dark-field illumination using a 5 × objective lens (MPlan FL N × 5; Olympus; *NA* = 0.15). The exposure times were set to 610 μs and 12.0 ms for bright-field and dark-field illumination, respectively. The LED power and contrast parameters of the cellSens software were set to 50% and “Low,” respectively.

### Confocal Imaging and Cell Segmentation

The plasma membranes labeled with GFP-PIP2a were captured with a microscope equipped with an objective lens (UPlanXApo × 20; Olympus; NA = 0.80), a spinning disk confocal scanning unit (CSU-W1; Yokogawa, Tokyo, Japan), a laser illumination homogenization unit (Uniformizer; Yokogawa), and a complementary metal-oxide semiconductor camera (Zyla; Andor, Belfast, United Kingdom). Cell segmentation was performed with the ImageJ plugin Morphological Segmentation (Legland et al., 2016). When the segmented images had apparent errors, the images were manually corrected. The assignment of cells between a manually traced image and the segmented image was performed manually using ImageJ.

### Measurement of Cotyledon Area

The cotyledon was set as horizontal to the focal plane as possible, and then the whole cotyledons were captured using a 5 × objective lens (MPlan FL N × 5; Olympus; *NA* = 0.15). The microscopic images were blurred with a Gaussian filter (sigma = 3 pixels) to reduce noise. Thereafter, the images were binarized with manual thresholding to define the cotyledon region. The cotyledon area in the binarized image was measured using ImageJ (Schneider et al., 2012).

### Image Processing for Morphometry of Cotyledon Epidermal Cells

To capture the entire cotyledon, omnifocal images taken at multiple positions were automatically stitched based on normalized cross-correlation using the ImageJ plugin LPX-Registration^1^ (Nagata et al., 2016; Supplementary Figure 1). For cell segmentation, we used the deep learning-based 2-D segmentation function of the image analysis software AIVIA (DRVision, Bellevue, WA, United States). First, we manually segmented the cell contours in 10 metallographic images using the ImageJ software. Then, the raw metallographic images and the manually segmented binary images were used as training data for AIVIA (Supplementary Figure 2). After training, the deep learning model output segmented images of cell contours from the input metallographic images. However, because the output image from the deep learning-based 2-D segmentation function of AIVIA was a grayscale image, not a binary image, the output image was binarized by threshold using ImageJ to determine the cell regions. Measurements of morphological features (cell area, perimeter, circularity, aspect ratio, and solidity) were performed using the “Analyze Particles” function in ImageJ (Schneider et al., 2012; Higaki et al., 2017; Kikukawa et al., 2021). Identification of the same cells in the time-lapse images [5– and 8-days after sowing (DAS)] was performed manually with ImageJ based on the cell shapes and locations. Regions where epidermal cells could not be captured even in the omnifocal images were excluded from the analysis. Circularity was defined as *4*π*SL*^–2^, where *S* and *L* represent the cell area and perimeter, respectively (Higaki et al., 2017; Kikukawa et al., 2021).

## Results

### Application of Silver Ink to Cotyledons and Metallographic Microscopy

**FIGURE 1:**
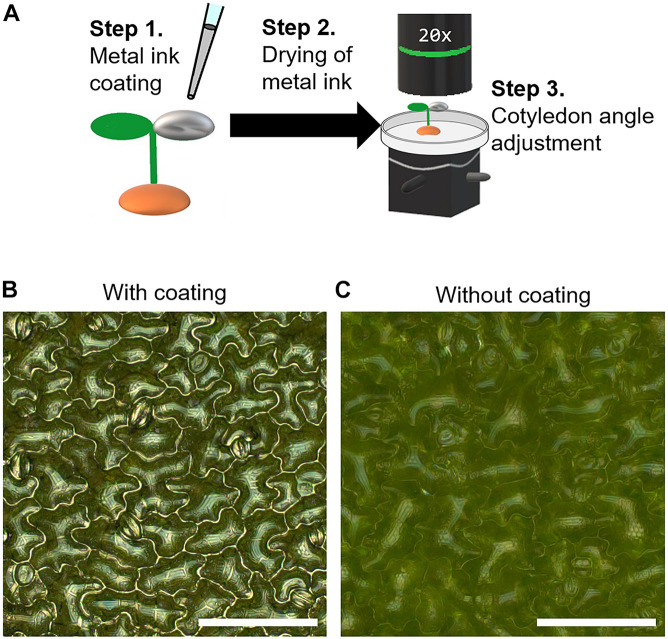
Silver ink coating of cotyledon surfaces for visualization of cotyledon epidermal cells in *Arabidopsis thaliana*. **(A)** Schematic illustration of the metal ink coating process. Step 1: The adaxial surface of a cotyledon of a 5-day-old *Arabidopsis thaliana* seedling was coated with a 0.2-μl drop of silver ink solution. Step 2: The seedling with the ink-coated cotyledon was kept for 30 min at room temperature to allow the silver ink solution to dry. Step 3: The cotyledon angle was adjusted using a biaxial goniometer so that the cotyledon was as horizontal as possible to the focal plane. **(B,C)** Omnifocal metallographic images of the 5-day-old *A. thaliana* cotyledons with **(B)** and without **(C)** the silver ink coating. Scale bars indicate 100 μm.

Before applying the silver ink solution, the tilt of the seedling was adjusted using tweezers so that the cotyledon was as horizontal as possible to prevent the silver ink from flowing onto the other cotyledon. Then, 0.2 μl of silver ink solution (0.31 wt%) was dropped onto the adaxial surface of an *A. thaliana* cotyledon at 5 DAS (Figure 1A, Step 1). The seedlings were kept for 30 min at room temperature to allow the silver ink solution to dry (Figure 1A, Step 2). To observe the cotyledon surfaces, the seedlings were carefully transferred to a Petri dish lid (φ40 × 13.5 mm) using tweezers with the culture soil still attached to the roots. The petri dish lid with the seedlings was placed on a biaxial goniometer, and each cotyledon was observed by adjusting the angle to be as horizontal as possible to the focal plane (Figure 1A, Step 3). When the surfaces of the silver ink-coated cotyledons were observed under a metallographic microscope, the epidermal cell contours were clearly observed (Figure 1B), whereas for the cotyledons with no silver ink coating, the cell shapes were unclear (Figure 1C).

### Illumination Conditions for the Metallurgical Microscope

**FIGURE 2:**
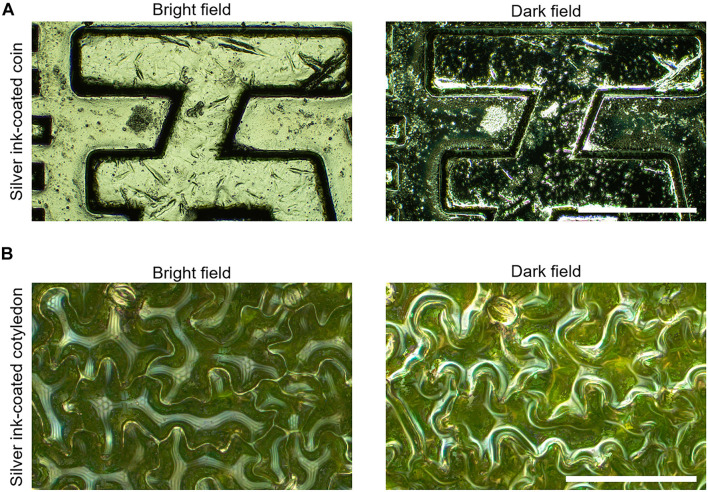
Evaluation of illumination methods for visualizing cotyledon epidermal cell contours by metallographic microscopy. **(A)** Omnifocal metallographic images of the surface of the silver ink-coated coin captured with bright-field (left) and dark-field (right) illumination. Scale bar indicates 500 μm. **(B)** Omnifocal metallographic images of the surface of a cotyledon coated with silver ink captured with bright-field (left) and dark-field (right) illumination. Scale bar indicates 100 μm. The surface microstructure of both the coin and the cotyledon surface was better observed with the bright-field illumination than with the dark-field illumination.

To determine the best illumination conditions for metallographic microscopy, we used the silver ink-coated coin as a standard sample and observed it under bright-field and dark-field illumination. For omnifocal metallographic images, we found that bright-field illumination was better than dark-field illumination for seeing fine scratches on the coin surface (Figure 2A). For the silver ink-coated cotyledons observed under the same lighting conditions, the epidermal cell shape was clearly visible in bright-field illumination, but obscured in dark-field illumination (Figure 2B). Therefore, all subsequent observations were performed in a bright field.

### Applicability of the Silver Ink Coating Method to Other Plant Species

To examine the wider applicability of the silver ink coating method, we applied silver ink to the cotyledons of non-model plants, carrot, petunia, Japanese white radish, and snap dragon, using the method described above for *A. thaliana*. The contours of the cotyledon epidermal cells were clearly observed in these plants (Supplementary Figure 3).

We also tested whether the proposed method would work for true leaves in *A. thaliana*. Simply dropping silver ink as we did for the cotyledons resulted in uneven application, probably because the trichomes prevented the silver ink from spreading on the leaf surface (Supplementary Figure 4A). However, by mechanical spreading the silver ink on the leaf with a micro spatula, we were able to observe the epidermal cell shape in a wide area of the true leaf as well as we did for the cotyledon (Supplementary Figure 4B).

### Validation of Cell Shape Quantification With Metallographic Images

**FIGURE 3:**
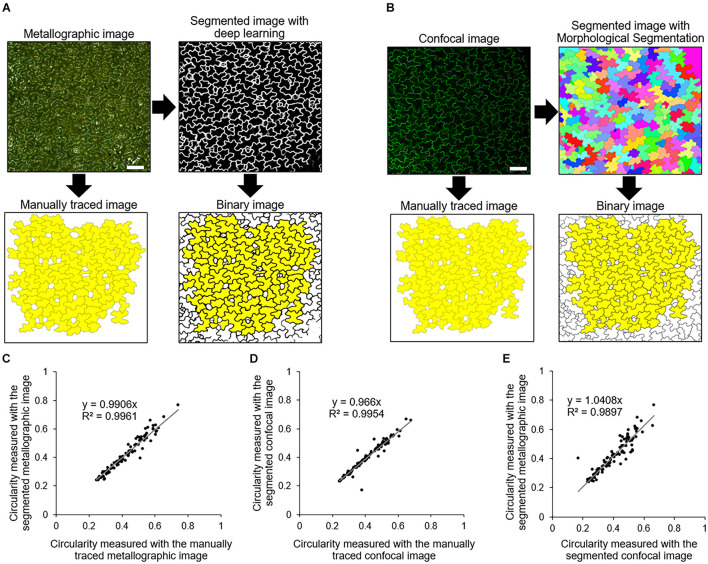
Cell segmentation and validation. **(A)** Manual tracing and segmentation based on deep learning of a metallographic image of a silver ink-coated *A. thaliana* cotyledon. Yellow indicates the cells that were used for measurements of cell circularity. *N* = 100. Scale bar indicates 100 μm. **(B)** Manual tracing and segmentation using Morphological Segmentation (Legland et al., 2016) of confocal images of a plasma membrane marker GFP-PIP2a. Yellow indicates the cells that were identical to those highlighted in yellow in **(A)**. Scale bar indicates 100 μm. **(C,D)** Scatter plots of the circularity of the cells highlighted in yellow in **(A,B)**, respectively. The relationships between the values measured with the manually traced images and the segmented images are shown. **(E)** Scatter plot of the circularity of the cells highlighted in yellow in the binary image in **(A,B)**.

To quantitatively evaluate cell shapes from metallographic images, we performed cell segmentation using a deep learning approach. To evaluate the accuracy of the deep learning-based cell segmentation, we compared it with the established segmentation method for confocal images that is commonly used to analyze epidermal cell shape (Legland et al., 2016). The cotyledons of transgenic *A. thaliana* stably expressing the plasma membrane marker GFP-PIP2a were coated with silver ink and observed with a metallographic microscope (Figure 3A, Metallographic image). Immediately after the acquisition of the metallographic images, the silver ink-coated cotyledons were observed with a confocal microscope, which allowed us to obtain a confocal image of the same cells as those captured with the metallographic microscope (Figure 3B, Confocal image). The epidermal cells in the confocal images were segmented using the ImageJ plugin Morphological Segmentation (Legland et al., 2016), and the cell circularity, which is a basic cell shape indicator, was measured (Higaki et al., 2017; Kikukawa et al., 2021). The cell circularity values measured from the segmented confocal images were highly correlated with the values measured from the manually traced images (decision coefficient *R*^2^ = 0.9954) (Figures 3B,D). Likewise, the cell circularity values measured with the deep learning-based segmented metallographic images were highly correlated with the values measured from the manually traced images (decision coefficient *R*^2^ = 0.9961) (Figures 3A,C), suggesting that the deep learning-based cell segmentation detected the epidermal cell contours in the metallographic image, as accurately as the established confocal image segmentation. Furthermore, the correlation between the values measured with the segmented confocal and metallographic images was high (decision coefficient *R*^2^ = 0.9897) (Figure 3E), suggesting that the proposed method with silver ink coating provided almost the same results as those obtained with the confocal microscope.

### Effects of Silver Ink Coating on *A. thaliana* Cotyledon Growth

**FIGURE 4:**
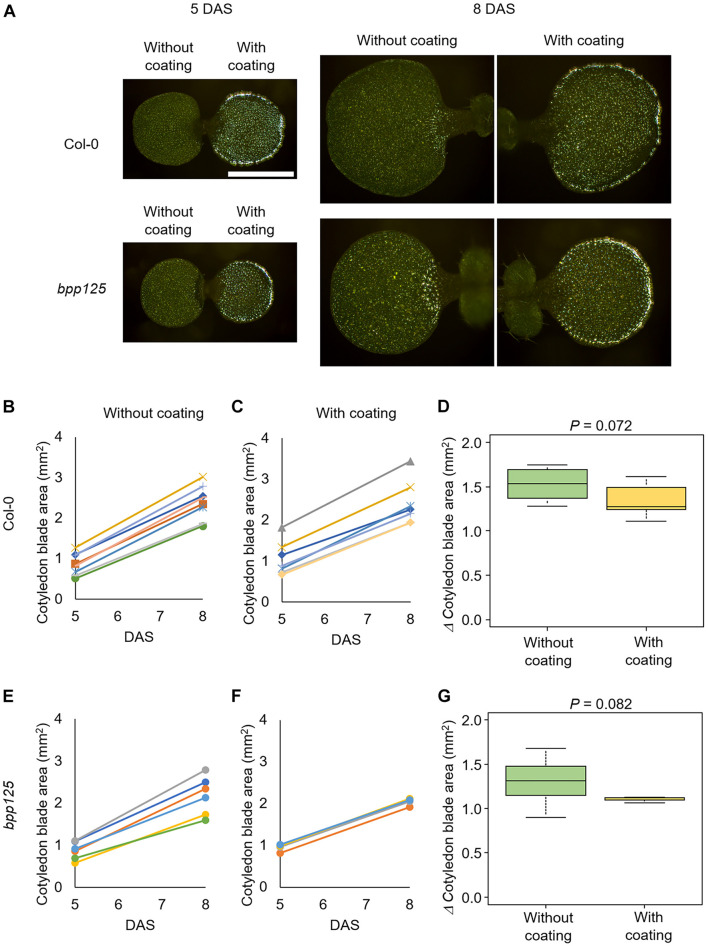
Effects of silver ink coating on the expansion growth of cotyledons. **(A)** Growth of seedlings with only one side of one of the cotyledons coated with silver ink. Silver ink was applied to one cotyledon of wild type (Col-0) (top) and *bpp125* triple mutant (bottom) seedlings. Growth was measured just after coating [5 days after sowing (DAS)] and again 3 days later (8 DAS). Both the silver ink-coated and uncoated cotyledons showed expanded growth. Scale bar indicates 1 mm. **(B–G)** Measurements of cotyledon blade area. The areas of wild type (Col-0) **(B,C)** and *bpp125* **(E,F)** cotyledons without **(B,E)** and with silver ink coating **(C,F)** were measured. The same marker in the graph indicates the same cotyledon, and thus the lines in **(B–F)** show the expanded growth of the same cotyledons. Changes in cotyledon blade area from 5 to 8 DAS in the wild type (Col-0) **(D)** and *bpp125* **(G)**. *P*-values were determined by Mann-Whitney’s *U*-test (*N* = 5–8).

To examine the effect of silver ink coating on cotyledon expansion growth, the silver ink solution was applied to one side of the cotyledons of wild-type (Col-0) and *bpp125* triple mutant seedlings at 5 DAS (Figure 4A, right cotyledon); the other cotyledon was untreated as a control (Figure 4A, left cotyledon). In both Col-0 and *bpp125*, the cotyledon area growth in the uncoated and coated seedlings was comparable for at least 3 days after the coating (Figures 4B–D). The effect of silver ink coating on cotyledon expansion growth was evaluated using by Mann-Whitney’s *U*-test. The *P*-values were found to be higher than the standard significance level [*P* = 0.072 (Col-0), 0.082 (*bpp125*)], which indicated that the inhibitory effect of silver ink coating on cotyledon growth was limited.

### Time-Lapse Analysis on Cell Morphology in the *bpp125* Triple Mutant

**FIGURE 5:**
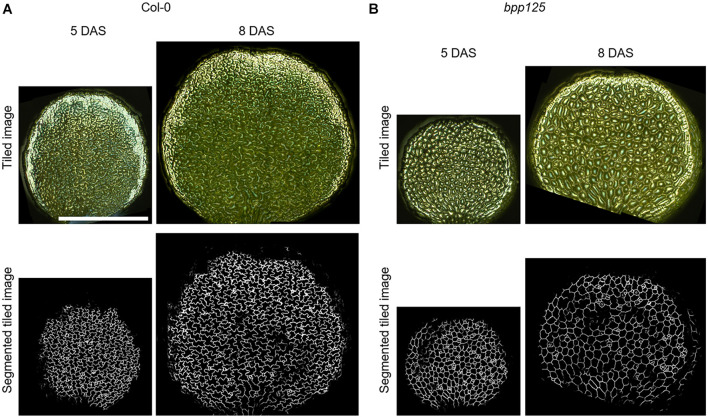
Wide-area cotyledon surface images and the deep learning-based epidermal cell segmentation from tiled omnifocal metallographic images. Tiled omnifocal metallographic microscopic images and segmented images obtained based on deep learning of wild type (Col-0) **(A)** and *bpp125* triple mutant **(B)** cotyledons. Growth was measured just after silver ink coating [5 days after sowing (DAS)] and again 3 days later (8 DAS). Scale bar indicates 1 mm.

**FIGURE 6:**
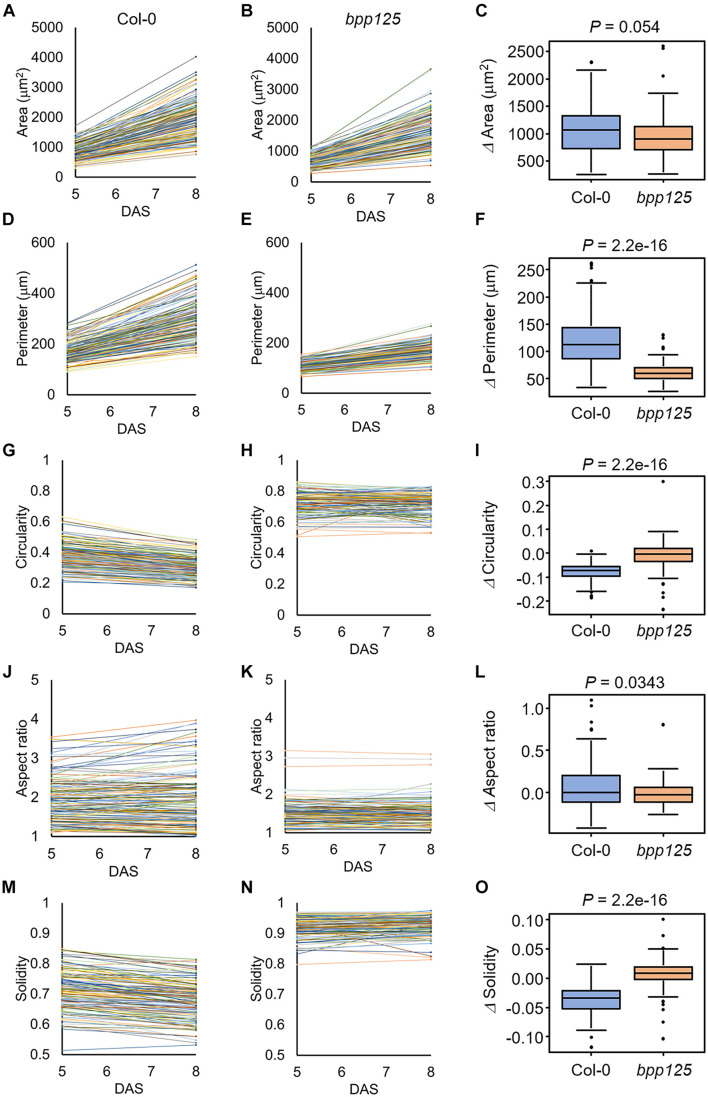
Measurements of morphological features of cotyledon epidermal cells. The results were obtained from 144 [wild type (Col-0)] and 119 (*bpp125* triple mutant) cells from nine independent seedlings at 5 and 8 days after sowing (DAS). **(A,B)** Time-course of cell area in the wild type (Col-0) **(A)** and *bpp125* **(B)**. **(C)** Changes in cell area. **(D,E)** Time-course of cell periphery length in the wild type (Col-0) **(D)** and *bpp125* **(E)**. **(F)** Changes in cell periphery length. **(G,H)** Time-course of cell circularity in the wild type (Col-0) **(G)** and *bpp125* **(H)**. **(I)** Changes in cell circularity. **(J,K)** Time-course of cell aspect ratio in the wild type (Col-0) **(J)** and *bpp125* **(K)**. **(L)** Changes in cell aspect ratio. **(M,N)** Time-course of cell solidity in the wild type (Col-0) **(M)** and *bpp125* (N). **(O)** Changes in cell solidity. *P*-values were determined by Mann-Whitney’s *U*-test (*N* = 119–144).

To demonstrate that our proposed method can be used to evaluate morphological changes of cotyledon epidermal cells, we measured the morphological features of growing cells in wild type and *bpp125* triple mutant seedlings (Wong et al., 2019). To capture the entire cotyledon, we stitched the omnifocal images taken at multiple positions (Supplementary Figure 1) and performed automatic cell segmentation based on deep learning from stitched omnifocal images (Figure 5). Then, using the segmented images, we measured the morphological features of epidermal cells in wild-type (Col-0) and *bpp125* mutant cotyledons coated with silver ink at 5 DAS (immediately after coating) and 8 DAS (3 days after coating) (Figure 6).

We found that the epidermal cell area was significantly increased in both the wild type and *bpp125* cotyledons (Figures 6A,B), which was consistent with the measurements of cotyledon area (Figures 4C,F). The difference in the increase in cell area after 3 days in the wild type and *bpp125* was not significant (*P* = 0.054) (Figure 6C). These results suggest that the epidermal cell area expanded in both the wild type and *bpp125*; however, the increase in cell perimeter in *bpp125* was significantly lower than it was in the wild type (Figures 6D–F).

Circularity is a measure of the length of the cell periphery per unit cell area (see section “Materials and Methods” for the definition). The highest value of 1 indicates a perfect circle, whereas the lowest value of 0 indicates a highly complex shape. The circularity measurements showed that most cells in the wild type had negative values for the amount of change, indicating the cell shapes became more complex as the plants grew (Figures 6G,I). Conversely, no significant change was observed in *bpp125* cells, suggesting that the balance between cell periphery length and cell area was maintained as the plants grew (Figures 6H,I).

The aspect ratio is the ratio of the length of the major axis to the length of the minor axis of a fitted ellipse of a cell, and this ratio is used as an index of cell elongation. The smallest value of 1 is obtained when the approximate ellipse is a perfect circle, and the value increases as the cell elongates (Higaki et al., 2017). The aspect ratio measurements showed no significant change in either the wild type or *bpp125*, suggesting that there was no significant change in cell elongation (Figures 6J–L).

Solidity is the ratio of cell area to the area of the convex hull of the cell and this ratio is used as an index of cell interdigitation. It reaches a maximum value of 1 when there is no waving in the lateral cell wall and approaches a minimum value of 0 when the lateral cell wall waving becomes more pronounced (Higaki et al., 2017). The solidity measurements showed a high percentage of cells in the wild type had decreased values, indicating that the cells became interdigitated (Figures 6M,O). Conversely, no significant change was observed in *bpp125* cells, suggesting that almost no waving occurred in the lateral cell wall (Figures 6N,O).

## Discussion

### Limitations and Strength of the Proposed Method

We proposed a novel method for monitoring the morphogenesis of *A. thaliana* cotyledon epidermal cells by coating the cotyledon surfaces with silver ink for metallographic microscopy. A limitation of this method is that it cannot provide three-dimensional shape information for cells because it captures only the cotyledon surface and is based on a two-dimensional approximation. To obtain three-dimensional structures of epidermal cells and their temporal changes (i.e., four-dimensional information), conventional confocal microscopy-based methods can be used (Wong et al., 2019; Haas et al., 2020; Higaki and Mizuno, 2020; Eng et al., 2021).

It was also difficult to observe epidermal cells in the region of the cotyledon margin using our method (Figure 5), probably because cells that are tilted to the focal plane are difficult to observe. Therefore, established techniques, such as confocal observation, still need to be used to capture margin cells (Bilsborough et al., 2011; Mansfield et al., 2018).

Although, under our conditions, the application of silver ink did not affect the *A. thaliana* cotyledon expansion (Figure 4), we cannot rule out the possibility of toxicity depending on the concentration of silver ink or the plant species. We used silver ink at a concentration of 0.31 wt%, which was determined to be the minimum concentration at which cell contours could be clearly visualized under our metallographic microscopy conditions, and automatic cell segmentation was achieved with high accuracy by deep learning (Figure 3).

Despite these limitations, a technical advantage of our proposed method is that it can be performed easily with low cost because metallographic microscopy is inexpensive and easy to use. More importantly, this method does not require transformation using fluorescent protein marker gene constructs but still allows accurate evaluation of cell shapes (Figure 3). This advantage will be useful in the analysis of non-model plants for which transformation methods have not yet been established (Supplementary Figure 3).

### Tips for Applying Silver Ink and Re-applying for Long-Term Cell Tracking

Although our proposed method is simple and easy to reproduce, application of the metal ink drop requires technical attention. Application of a 0.2-μl drop of metal ink onto the axial side of the cotyledon surface is a simple process, but if the cotyledon is tilted the drop may run onto the shoot meristem or neighboring cotyledon. To prevent this problem, seedlings with cotyledons that are fully open and at a horizontal angle to the ground should be selected. Alternatively, the entire seedling can be tilted so that the tip of the cotyledon faces slightly downward before applying the metal ink. In our experimental system, the variation of cotyledon area at 5 DAS was not large (Figures 4B,C,E,F); therefore, we uniformly applied 0.2 μl of silver ink solution to all cotyledons. However, depending on the target area for analysis, it may be possible to vary the amount applied so that the density of metal ink remains constant.

We demonstrated the usefulness of the proposed method for time-lapse analysis using the *bpp125* triple mutant, which has been reported to show abnormal morphology of cotyledon pavement cells (Wong et al., 2019; Figures 5, 6). The pavement cell morphology of *bpp125* was previously evaluated using excised cotyledons from plants at different ages (Wong et al., 2019), and, in this study, we were able to track the same cells and measure changes in morphological features (Figure 6). Our measurements directly showed that most *bpp125* pavement cells, unlike those of the wild type, grew isotopically with a constant cell morphology as evaluated by circularity, aspect ratio, and solidity from 5 to 8 DAS (Figure 6).

We coated the 5-DAS cotyledon with the silver ink and successfully observed the epidermal cell contours 3 days after coating (8 DAS) (Figure 5). However, after approximately 5 days (10 DAS), the intensity of the reflected light became weaker, probably because of thinning of the silver ink caused by cotyledon expansion (Supplementary Figures 5A,B). We confirmed that the cells could still be clearly visualized by re-coating 5 days after the first coating (10 DAS) (Supplementary Figure 5C). Of course, re-coating of silver ink is laborious and time-consuming, and it is also necessary to re-examine the effects on cotyledon expansion (Figure 4) to decide when to re-coat. Nevertheless, re-coating could be useful for long-term cell shape tracking.

## Data Availability Statement

The raw data supporting the conclusions of this article will be made available by the authors, without undue reservation.

## Author Contributions

TH participated in the design of the study and wrote the manuscript. KK, KY, and TH analyzed the data. KK assisted in revising the manuscript. All authors performed the experiments, read, and approved the final version of the manuscript.

## Conflict of Interest

The authors declare that the research was conducted in the absence of any commercial or financial relationships that could be construed as a potential conflict of interest.

## Publisher’s Note

All claims expressed in this article are solely those of the authors and do not necessarily represent those of their affiliated organizations, or those of the publisher, the editors and the reviewers. Any product that may be evaluated in this article, or claim that may be made by its manufacturer, is not guaranteed or endorsed by the publisher.

## Footnotes

1
https://lpixel.net/products/lpixel-imagej-plugins/

## Supplementary Material

Supplementary Figure 1 Acquisition of wide-area cotyledon surface images. **(A)** Representative multi-point omnifocal images. **(B)** Tiled image of the images shown in **(A)**. The wide-area cotyledon surface image was reconstructed by automatic tiling of multi-point images. Scale bar indicates 400 μm.

Supplementary Figure 2 Representative images of the training data for deep leaning-based cell segmentation. Using the grayscale raw input image (left), the cell contours were manually traced (right). These image sets were used for training the image analysis software AIVIA.

Supplementary Figure 3 Application of silver ink coating for non-model plants. Silver ink-coated cotyledons of 7-day-old seedlings of carrot (*Daucus carota*), petunia (*Petunia hybrida*), Japanese white radish (*Raphanus sativus*), and snapdragon (*Antirrhinum majus*) are shown. Scale bars indicate 100 μm.

Supplementary Figure 4 Application of silver ink coating for true leaves of *Arabidopsis thaliana*. Silver ink-coated rosette true leaves of 31-day-old *A. thaliana* are shown. **(A)** Representative image of a true leaf with a drop of silver ink, as was done for the cotyledons. **(B)** Representative image of a true leaf with drop and spread of silver ink with a micro spatula. The epidermal cell contours were visualized over trichomes by mechanical spreading the ink. Scale bars indicate 100 μm.

Supplementary Figure 5 Re-coating of silver ink. **(A)** Omnifocal metallographic image of the cotyledon surface of seedling of *A. thaliana* 5 days after sowing (DAS). **(B)** Omnifocal metallographic image of the same area as in **(A)** after 5 days (10 DAS). **(C)** Omnifocal metallographic image in which silver ink was re-coated just after capturing the image in **(B)**. The same region was observed again. The cell contour signal was stronger after the re-coating.
