# Supplementary material for: Metal-Nano-Ink Coating for Monitoring and Quantification of Cotyledon Epidermal Cell Morphogenesis
Source: Front Plant Sci. 2021 Sep 21;12:745980. doi: 10.3389/fpls.2021.745980 (PMC8490765; doi:10.3389/fpls.2021.745980)
Supplement: Supplementary Figure 1 — Acquisition of wide-area cotyledon surface images. (A) Representative multi-point omnifocal images. (B) Tiled image of the images shown in (A). The wide-area cotyledon surface image was reconstructed by automatic tiling of multi-point images. Scale bar indicates 400 μm. [file Data_Sheet_1.PDF]

Supplementary Figures

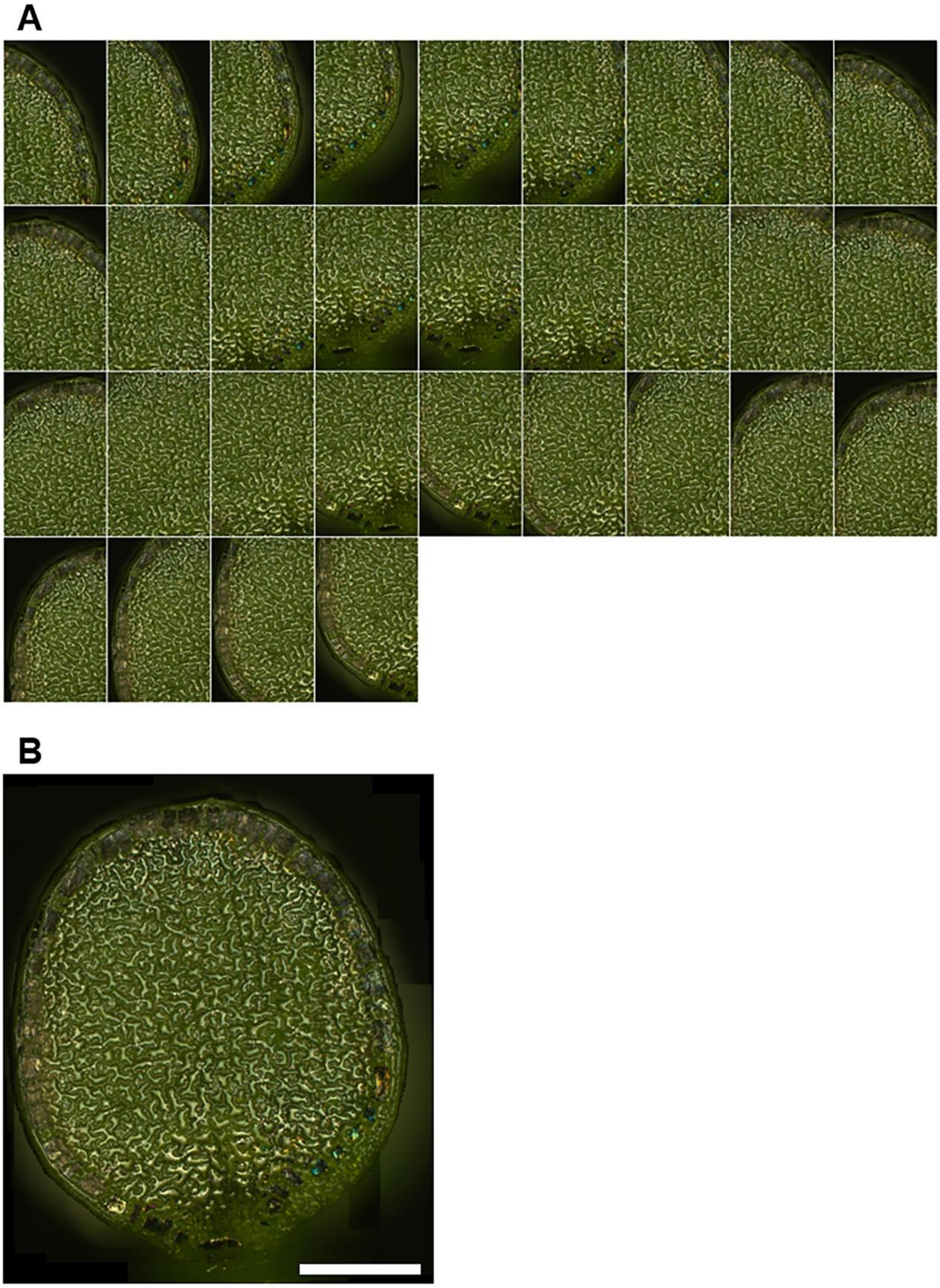

Supplementary Figure 1. Acquisition of wide-area cotyledon surface images. (A) Representative multi-point omnifocal images. (B) Tiled image of the images shown in (A). The wide-area cotyledon surface image was reconstructed by automatic tiling of multi-point images. Scale bar indicates 400 µm.

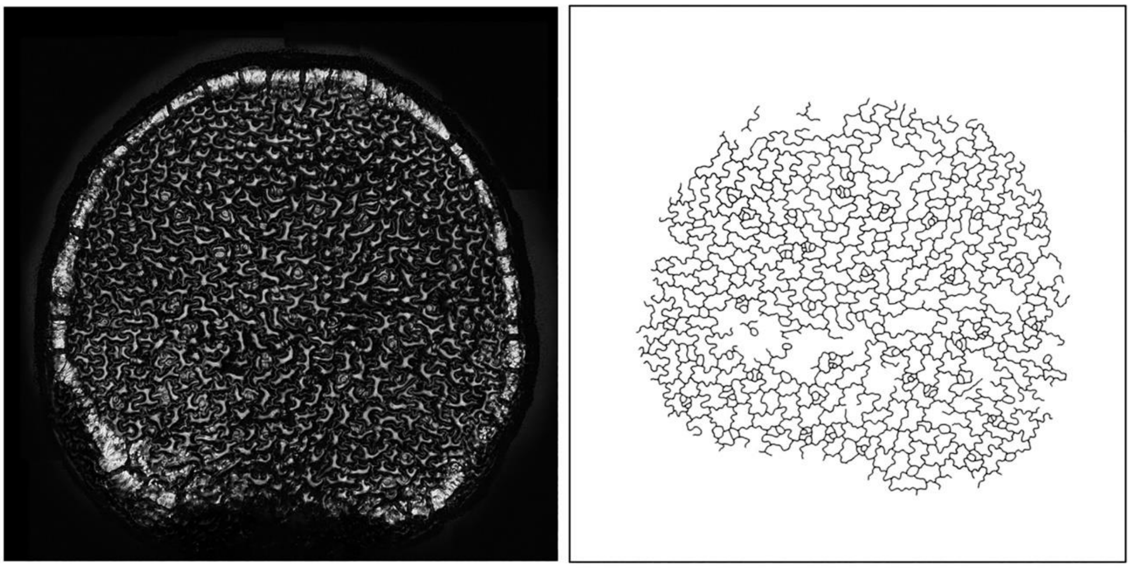

Supplementary Figure 2. Representative images of the training data for deep leaning-based cell segmentation. Using the grayscale raw input image (left), the cell contours were manually traced (right). These image sets were used for training the image analysis software AIVIA.

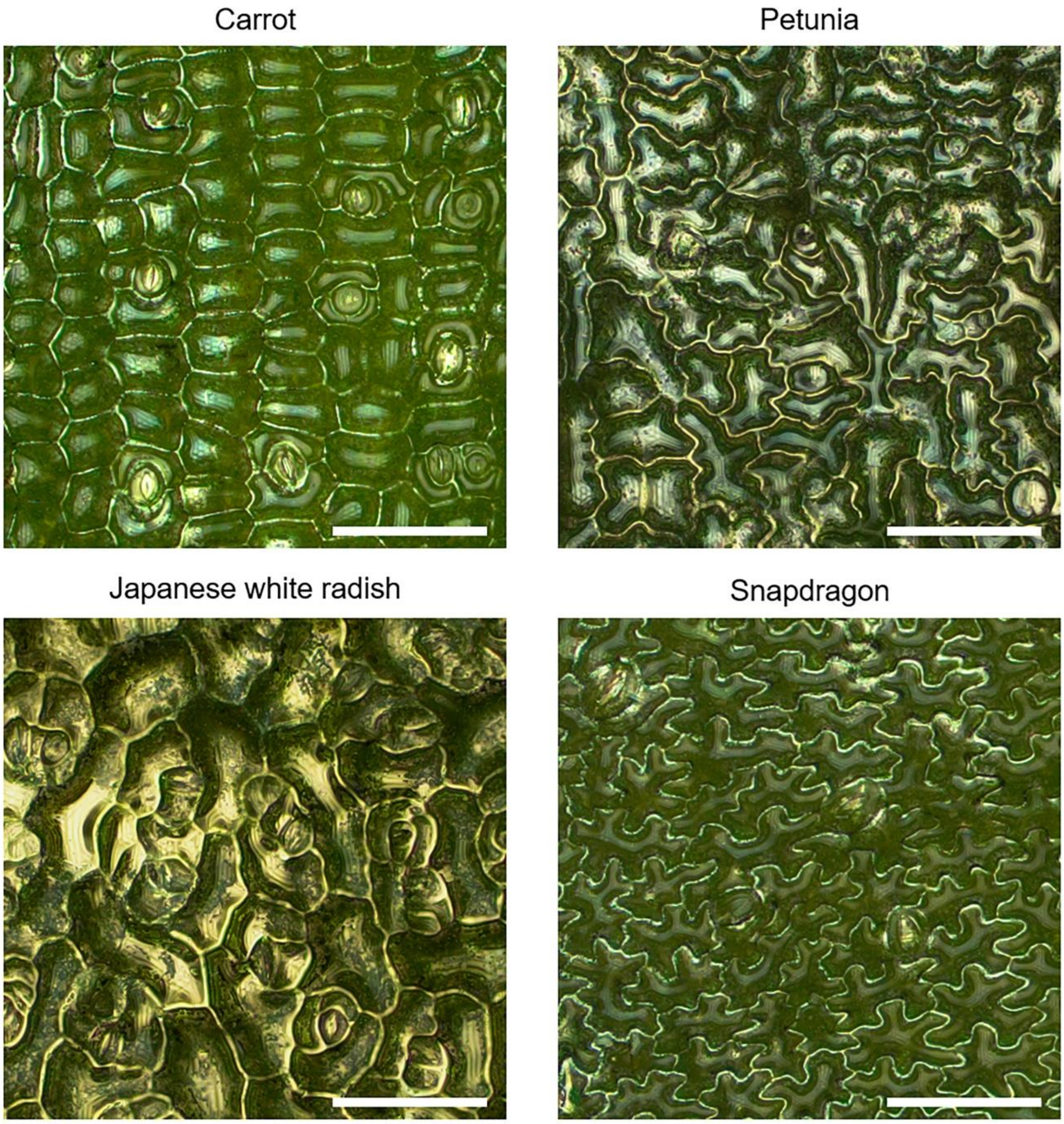

Supplementary Figure 3. Application of silver ink coating for non-model plants. Silver ink-coated cotyledons of 7-day-old seedlings of carrot (*Daucus carota*), petunia (*Petunia hybrida*), Japanese white radish (*Raphanus sativus*), and snapdragon (*Antirrhinum majus*) are shown. Scale bars indicate 100 µm.

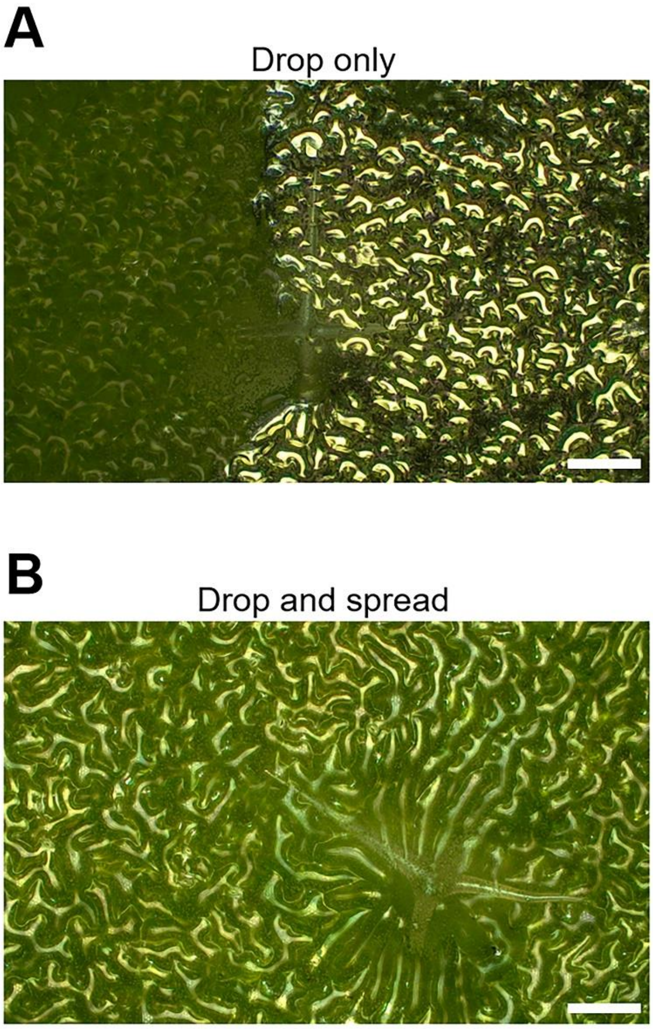

Supplementary Figure 4. Application of silver ink coating for true leaves of *Arabidopsis thaliana*. Silver ink-coated rosette true leaves of 31-day-old *A. thaliana* are shown. (A) Representative image of a true leaf with a drop of silver ink, as was done for the cotyledons. (B) Representative image of a true leaf with drop and spread of silver ink with a micro spatula. The epidermal cell contours were visualized over trichomes by mechanical spreading the ink. Scale bars indicate 100 μm.

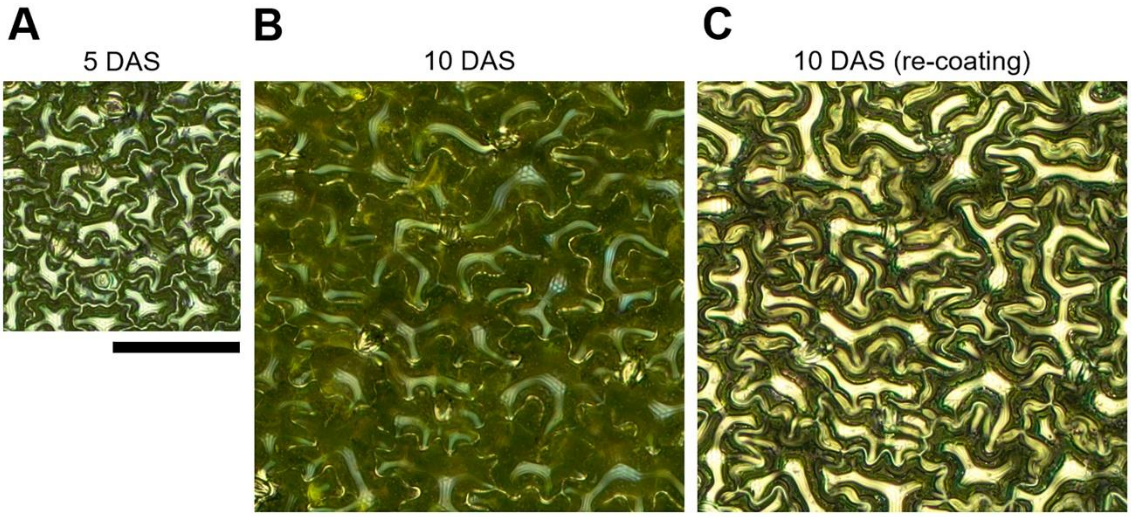

Supplementary Figure 5. Re-coating of silver ink. (A) Omnifocal metallographic image of the cotyledon surface of seedling of *A. thaliana* 5 days after sowing (DAS). (B) Omnifocal metallographic image of the same area as in (A) after 5 days (10 DAS). (C) Omnifocal metallographic image in which silver ink was re-coated just after capturing the image in (B). The same region was observed again. The cell contour signal was stronger after the re-coating.
